# Editorial: Facilitating the Third Wave of Positive Psychology: Perspectives on the Future of the Discipline

**DOI:** 10.3389/fpsyg.2022.963167

**Published:** 2022-07-01

**Authors:** Llewellyn E. van Zyl, Marisa Salanova

**Affiliations:** ^1^Department of Industrial Engineering and Innovation Sciences, University of Eindhoven, Eindhoven, Netherlands; ^2^Optentia Research Unit, North-West University, Vanderbijlpark, South Africa; ^3^Department of Human Resource Management, University of Twente, Enschede, Netherlands; ^4^Department of Social Psychology, Institut für Psychologie, Goethe University, Frankfurt am Main, Germany; ^5^WANT Research Team, Department of Developmental Psychology, Education, Social Psychology and Methodology, Universitat Jaume I, Castelló de la Plana, Spain

**Keywords:** third wave positive psychology, positive psychology, future perspectives, research agendas, positive psychological interventions

## Introduction

The science of positive subjective experiences, positive characteristics (states/traits/behaviors), and positive institutions have shaped our collective understanding of the elements that make life worth living and the factors that improve- and distract from optimal functioning (Donaldson et al.; Ng et al., 2021). Positive psychology has spawned a magnitude of new approaches, theories and methodologies that not only explained the conditions required for optimal functioning but also proved to be useful in many adjacent fields such as organizational studies, education, health, risk management, and even architectural sciences (Van Zyl and Rothmann, 2022). This, in turn, resulted in the creation of various new research and practice domains ranging from positive artificial intelligence (da Silva, 2020) and positive computing (Jeong et al., 2020) to positive coaching (Van Zyl et al., 2020; Richter et al., 2021) workplace design, human-robot collaboration and positive design sciences (Van Zyl and Rothmann, 2022). The science of understanding “what is right” rather than “what is wrong” has thus produced significant insights into the human condition and provided practical solutions to complex societal problems (Ryff; Worthington and Van Zyl). The discipline, however, isn't stagnant and continues to grow and develop. Lomas et al. (2021) stated that the discipline is on the verge of a third wave of innovative research that will fundamentally alter the trajectory of its discourse. However, it's not yet clear what the future of positive psychology will hold? What are some of the grand challenges facing this third wave of positive psychological research? Moreover, how can we, as a collective, contribute to enhancing the credibility and impact of the discipline's future? These are some of the most challenging questions that need to be answered. With the rapid development of the field, detailed research and practice “roadmaps” are thus required to set a course for future perspectives in positive psychology.

This Research Topic aimed to address these questions by collating a series of research agendas about potential future perspectives in positive psychology. Specifically, this Research Topic aimed to identify the limitations in our current understanding of the different theories, models, methods and interventions on which positive psychology is built and propose a roadmap for the third wave of Positive Psychological Research.

## Structure and Contribution of The Research Topic

Through the collective efforts of some of the top minds in our field today, this Research Topic provides 19 visionary, inspiring and provocative ideas about the future of our discipline. The manuscripts in this Research Topic, summarized in Table 1, are classified into four sections:

1. *Future Perspectives on Positive Psychology as a Discipline*. In this section, the authors reflect upon the growth of the discipline as a whole, highlight various challenges it faces and present a vision for how the discipline could develop.
2. *Future Perspectives on Mental Health and Wellbeing*. Here, the focus was on presenting new models and integrated approaches related to conceptualizing, measuring and managing mental health and wellbeing in various life domains.
3. *Future Perspectives on Specific Positive Psychological Constructs*. In this section, the authors provided new perspectives on popular positive psychological constructs, conceptualized new theories and approaches, and provided reflections on how future researchers could take these forward.
4. *Future Perspectives on Positive Psychological Interventions*. The final section aimed to provide new frameworks or typologies relating to the development and implementation of positive psychological interventions in various life domains. Specifically, it aimed to provide guidelines on designing, implementing and evaluating highly effective interventions and what approaches may be helpful in the future.

**Table 1:** Summary of future perspectives on positive psychology.

**No**	**Author**	**Title**	**Purpose**	**Views**	**Citations**
*Future perspectives on positive psychology as a discipline*					
1	Ryff	Positive Psychology: Looking Back and Looking Forward	To reflect upon the past criticisms, present problems and future perspectives of positive psychology as a discipline.	1,350	1
2	Wissing	Beyond the “Third Wave of Positive Psychology”: Challenges and Opportunities for Future Research	Reflecting upon the trends and nature of the “third wave” of positive psychology and provides a vision for positive psychology's future as an inter- or transdisciplinary science on wellbeing. The paper focused on unpacking the challenges, opportunities, and future perspectives of positive psychology as a discipline.	1,622	1
3	Joseph	How Humanistic Is Positive Psychology? Lessons in Positive Psychology From Carl Rogers' Person-Centred Approach—It's the Social Environment That Must Change	To discuss the relationship between- and call for further integration of positive psychology and humanistic psychology. Specifically, the aim was to highlight the importance of the social environment in facilitating personal growth and development.	8,394	3
*Future perspectives on mental health and wellbeing*					
4	Bohlmeijer and Westerhof	The Model for Sustainable Mental Health: Future Directions for Integrating Positive Psychology Into Mental Health Care	Presenting a model for sustainable mental health care by integrating positive psychological perspectives and different mental wellbeing approaches. This heuristic model provides a more balanced approach to developing, implementing, and evaluating mental health treatment strategies by incorporating both a complaint- and strength-oriented approach.	3,502	3
5	Donaldson et al.	PERMA+4: A Framework for Work-Related Wellbeing, Performance and Positive Organizational Psychology 2.0	Providing a more holistic approach to work-related wellbeing through expanding upon Seligman's (2012) PERMA Framework.	2,569	2
6	Duncan et al.	An Emerging Preventive Mental Health Care Strategy: The Neurobiological and Functional Basis of Positive Psychological Traits	Reflecting upon the relationship between positive psychological constructs (measured by self-report measures) and their relationship to neurological functions. Specifically, it provides guidelines for future research on measuring and managing positive psychological constructs and how these should be approached from a neurological perspective.	874	0
7	Vella-Brodrick et al.	Seeing Is Believing: Making Wellbeing More Tangible	To call for more objective or “tangible” measures for measuring and managing wellbeing. The paper provides current examples of “tangible” wellbeing and calls for more initiatives to advance this approach.	352	0
8	Weijers	Don't Miss the Well-Being Train: A Radical Proposal for Revolution in Positive Psychology	Calling for interdisciplinary perspectives on the conceptualization, consolidation, measurement and management of wellbeing.	683	0
*Future perspectives on specific positive psychological constructs*					
9	Bryant	Current Progress and Future Directions for Theory and Research on Savoring	To provide a snapshot of the current state of the art in savoring research and to present a roadmap for future research.	1,934	1
10	Colla et al.	“A New Hope” for Positive Psychology: A Dynamic Systems Reconceptualization of Hope Theory	To argue for an interdisciplinary approach to expanding the meta-theoretical, theoretical, and methodological horizons, enabling a more dynamic systems approach to study hope. Specifically, it provides a roadmap for alternative approaches and methodologies which could address limitations in contemporary hope research.	2,641	1
11	Peifer et al.	A Scoping Review of Flow Research	To provide a multilevel framework for flow which consists of three levels: (a) the individual, (b) the context and (c) the cultural level. In the first “Individual” level are the categories for personality, motivation, physiology, emotion, cognition, and behavior. The second “Contextual” level contains the categories for contextual and interindividual factors and the third “Cultural” level contains cultural factors related to flow. The authors expand upon their integrated model for flow and set an agenda for future research.	2,825	1
12	Schaufeli	Engaging Leadership: How to Promote Work Engagement?	To present a conceptual framework for “engaging leadership” and review the empirical work underpinning its construction. It integrates engaging leadership within the current positive organizational psychology lexicon and presents a roadmap for future research.	3,475	1
13	Vallerand et al.	The Role of Passion in Psychological and Cardiovascular Responses: Extending the Field of Passion and Positive Psychology in New Directions	Investigating the role of passion in physiological responses of individuals. Specifically, it aimed to investigate the role of passion, and the mediating role of cognitive appraisals, in the psychological- and physiological responses to stressful situations.	722	0
14	Wehmeyer	The Future of Positive Psychology and Disability	To summarize the factors that led to the emergence of a focus on the positive psychology of disability and a strength-based approach in the field, examine the state of knowledge and practice as it pertains to the positive psychology of disability, and examine the challenges that serve as barriers to progress in this area and opportunities for advancement.	1,184	1
*Future perspectives on positive psychological interventions*					
15	Ciarrochi et al.	Toward a Unified Framework for Positive Psychology Interventions: Evidence-Based Processes of Change in Coaching, Prevention, and Training	To present an integrated model for positive psychological processes through an extended evolutionary psychological perspective. Specifically, the paper postulates that a multi-dimensional and multilevel evolutionary approach could organize effective change processes in psychosocial interventions by focusing on context-appropriate variation, selection, and retention of processes, arranged in key biopsychosocial dimensions across psychological, biophysiological, and sociocultural levels of analysis.	7,320	0
16	Nielsen and Christensen	Positive Participatory Organizational Interventions: A Multilevel Approach for Creating Healthy Workplaces	To argue for more positive participatory organizational-level interventions to enhance wellbeing and performance. Specifically, the paper calls for more multi-leveled intervention approaches aimed at managing the job characteristics (demands/resources) of individuals, groups, leaders and organizations.	2,707	6
17	Passmore and Evans-Krimme	The Future of Coaching: A Conceptual Framework for the Coaching Sector From Personal Craft to Scientific Process and the Implications for Practice and Research	To provide a structured framework for the future development of coaching research.	4,421	0
18	Van Woerkom	Building Positive Organizations: A Typology of Positive Psychology Interventions	To develop an evidence-based typology for positive organizational interventions. The paper distinguishes between target level of organizational interventions (organization, group or individual) and between one-off and structural interventions. The paper concludes with suggestions on improving the long-term effectiveness of positive organizational interventions.	3,679	0
19	Worthington and Van Zyl	The Future of Evidence-Based Temperance Interventions	To provide an overview of the challenges and opportunities for expanding the theoretical conceptualization of temperance and reflect upon the challenges in temperance-related PPIs. The paper provides a research agenda for each aspect of temperance, and presents a roadmap for future temperance interventions.	2,172	1

## Future Perspectives and Research Agendas

Each of the 19 papers presents clear guidelines for taking the discipline forward and highlights the areas needed to build out positive psychology's relevance. Taking the overall trends of these papers together, this Research Topic highlights the following:

1. *Positive Psychology is not without its criticisms and critiques*. Joseph, Ryff, Wissing and others highlighted that despite positive psychology's rapid growth during the last two decades, there are still several challenges which need to be addressed. They argued that various critics have questioned the unique contribution of positive psychology as well as the validity of the theories, frameworks and models on which it is built. Ryff and Wissing highlighted the challenges relating to positive psychology's meta-theory, the questionable research practices positive psychologists employ, the validity of positive psychological assessments, the politics behind and exclusivity driving the discipline, the ineffectiveness of positive psychological interventions, its over-reliance on empiricism and that positive psychology is culturally biased. Joseph, on the other hand, argued that positive psychology lacks a holistic view of human nature and discussed the fragile relationship between humanistic psychology and positive psychology. These, and other authors, provided suggestions on how these could be addressed and how we can take the field forward.
2. *Positive Psychology requires more holistic, integrative and sustainable models for facilitating mental health and wellbeing*. Bohlmeijer and Westerhof, Donaldson et al., and Duncan et al. stated that more holistic approaches need to be developed in order to facilitate mental health development. Here the focus should be on integrating current perspectives of the individual with more dynamic approaches related to the impact of the environment and (social) context. These new approaches to mental health and wellbeing management should focus on developing the “strengths” of the individual *and* managing mental health complaints (Van Zyl et al., 2021). Further, environmental factors (such as workplace climate, workplace design, financial status, physical health etc.) should be incorporated into mental health models. More objective measures should also be used to make wellbeing more “tangible” (Vella-Brodrick et al.). Weijers also argued that sustainable mental health interventions and approaches require interdisciplinary perspectives and that the field should be opened up to incorporate these more actively.
3. *Positive psychology requires more multilevel approaches and multi-dimensional perspectives*. Various authors have highlighted the limitations of positive psychology's preference for understanding the individual in isolation from the environment (c.f. Ciarrochi et al.; Colla et al.; Nielsen abd Christensen; Peifer et al.). Individual behavior and experiences are not only a function of what is going on “within” the individual but also a result of what occurs “between” people and is influenced by the conditions of the environment in which they function. Further, positive psychology has been criticized for employing an oversimplified view of “positive institutions” by stating that positive institutional phenomena are nothing more than the aggregated mean experiences of individuals. More multi-leveled perspectives on positive states, traits and behaviors are required to address this limitation, especially when these are embedded within formal social systems such as organizations or communities.
4. *Positive Psychology and its relationship to physiological functioning*. Duncan et al., Vallerand et al., Vella-Broderick et al. and others have called for more empirical research on the relationship between positive psychological states, traits and behaviors and physical wellbeing/physiological factors/neurology. Although a significant amount of attention has been placed on understanding individuals' (self-reported) positive states and experiences, limited attention has been given to how these are related to physiological wellbeing and neurological functioning. There is thus a call for increased attention to the relationship between positive psychological constructs and physical health.
5. *Increasing the effectiveness and long-term effects of Positive Psychological Interventions*. Contributors to this Research Topic called for more attention on designing effective positive psychological interventions. Here, the focus should be on how positive psychological interventions should be designed to ensure sustainable changes in wellbeing over time. Positive psychological interventions that have been shown to work well should be dissected, and the reasons for their effectiveness unpacked. When interventions fail to produce the intended results, researchers should not look for contextual factors explaining the results away, but rather reflect on the methods and content of these interventions to determine why they did not work well. There is also a call for more alignment and standardization in positive psychological intervention design approaches and methodologies.
6. *New approaches to understanding positive psychological phenomena are required*. The COVID-19 pandemic showed that positive psychological approaches and theories cannot readily explain changes in behavior during a crisis. This implies that the usefulness of positive psychological theories, tools and techniques may only be applicable in relatively stable and predictable environments. Therefore, new approaches and theories are needed to help explain how positive states, traits, and behaviors can be facilitated in times of crisis.
7. *Developing more indigenous psychologies*. Positive psychology has been criticized for being a primarily western driven enterprise which is culturally biased. The current narrative of a one-size-fits-all approach to understanding positive states, traits and behaviors is not only culturally incensive but facilitates cultural biases and reinforces certain cultural stereotypes. As such, Wissing argued that more indigenous positive psychologies should be developed that are centered around the ideals, values, and world views of different cultures.
8. *Positive psychology needs to employ more robust research methods, designs and approaches*. Positive psychology is known for over-emphaisisng the importance of empiricism and positivism and over-relies on cross-sectional designs to support its claims. It has swayed away from using more robust methods such as qualitative research, experimental designs and mixed-method approaches. For positive psychology to grow, more robust research methods and approaches are required to describe relationships between factors but also to explain how/why these relationships exist.
9. *Positive Psychology should capitalize on rapid changing technologies*. Positive psychologists should be at the forefront of adopting new technological innovations to assess, develop, and distribute positive psychological tools and techniques. New developments in machine learning, natural language processing, augmented reality and digital meta-verses provide exciting new avenues for the discipline to grow.

## Conclusion

Positive psychology has shown to be the fastest-growing sub-discipline of psychology and has gained significant attention in practice (Martín-del-Río et al., 2021). It's rapid growth during the last two decades indicates that the discipline is on the horizon of a new wave of pioneering research, inspiring ideas and ground-breaking innovations. This new wave of research will be characterized by the rapid adoption of new technological innovations (e.g., artificial intelligence systems and machine learning), and will require more sophisticated models, approaches and measures to explain complex psychological phenomena. Facilitating the growth of the discipline will also require closer collaboration between scientists/practitioners, organizations/communities and professional societies/regulators to fast track the development and implementation of scientific innovations. We hope that this collection of articles from some of the top minds in our field, will inspire researchers to explore new opportunities and that it will provide a roadmap for future research.

## Author Contributions

LZ wrote the manuscript. MS co-managed the Research Topic and made conceptual contributions to the editorial. Both authors contributed to the article and approved the submitted version.

## Conflict of Interest

The authors declare that the research was conducted in the absence of any commercial or financial relationships that could be construed as a potential conflict of interest.

## Publisher's Note

All claims expressed in this article are solely those of the authors and do not necessarily represent those of their affiliated organizations, or those of the publisher, the editors and the reviewers. Any product that may be evaluated in this article, or claim that may be made by its manufacturer, is not guaranteed or endorsed by the publisher.
